# Leveraging Promotional Strategies to Enhance Hospital Influence on Social Media: Cross-Sectional Study

**DOI:** 10.2196/70676

**Published:** 2025-08-22

**Authors:** Xinyun Zhong, Yiyuan Ma, Haiyue Yu

**Affiliations:** 1 School of Communication Hong Kong Baptist University Hong Kong China (Hong Kong); 2 Institute of Applied Economics Shanghai Academy of Social Sciences Shanghai China; 3 Shanghai Municipal Hospital of Traditional Chinese Medicine Shanghai University of Traditional Chinese Medicine Shanghai China; 4 Shanghai Municipal Hospital Research Center for Disease Quality and Safety Shanghai China

**Keywords:** social media, influence, promotional strategies, hospitals, multivariate linear regression

## Abstract

**Background:**

Social media has become a vital platform for hospitals to engage with the public, disseminate health knowledge, and build trust. While promotional strategies have shown potential to enhance social media influence, the mechanisms through which these strategies impact hospital influence on social media remain unclear. Furthermore, the effectiveness of these strategies may vary across hospitals of different types and regions, necessitating a deeper understanding of their contextual applicability.

**Objective:**

On the basis of an integrated theoretical framework that combines multidisciplinary perspectives from communication, marketing, and organizational management, this study aimed to investigate the effects of 3 dimension-based promotional strategies (activity, interactivity, and entertainment value) on 4 dimensions of hospital social media influence (audience reach, audience approval, audience interaction, and dissemination power). It also sought to explore variations in the effectiveness of these strategies across hospitals of different types and regions.

**Methods:**

Data were collected from 721 officially verified hospital Weibo accounts and integrated with multidimensional hospital characteristics from the DXY National Hospital Database. Multivariate linear regression analysis was used to assess the relationship between promotional strategies and social media influence while controlling for hospital-specific characteristics. Heterogeneity analysis was conducted by incorporating interaction terms between promotional strategies and categorical dummy variables (used to group hospitals based on their characteristics) into the regression model. In addition, robustness checks were implemented to validate the stability of the main findings.

**Results:**

The promotional strategies of activity and interactivity demonstrated significant and consistent positive effects across all dimensions of social media influence. Specifically, a 10% increase in the number of Weibo posts (activity) was associated with a 4.8% increase in followers (*P*<.001), a 4.4% increase in maximum likes (*P*<.001), a 3.1% increase in maximum comments (*P*<.001), and a 4.9% increase in maximum shares (*P*<.001). Similarly, a 10% increase in the number of accounts followed by hospitals (interactivity) corresponded to a 4.5% increase in followers (*P*<.001), a 2.4% increase in maximum likes (*P*=.002), a 2.4% increase in maximum comments (*P*<.001), and a 3.1% increase in maximum shares (*P*<.001), whereas entertainment value had a more limited impact, significantly enhancing audience reach and approval in specific contexts. The heterogeneity analysis indicated that *hospital ownership* (public vs private), *size*, *ranking*, and *regional location* were key factors influencing the effectiveness of social media promotional strategies.

**Conclusions:**

This study provides empirical evidence on how promotional strategies influence hospital social media performance and underscores the importance of tailoring strategies based on hospital type and regional characteristics. The findings emphasize that hospitals should strategically choose communication approaches that align with their unique characteristics. By tailoring promotional strategies to their specific attributes and regional contexts, hospitals can effectively enhance their social media influence, foster stronger public trust, and promote sustainable digital transformation.

## Introduction

### Background

Social media has become a crucial bridge for hospitals to interact and communicate with the public in addressing rapid digital transformation issues. Disseminated health knowledge, shared medical information, and showcased professional services can also be provided [1-3]. Leveraging the extensive reach and immediacy of the internet, hospitals can use social media to establish close connections with patients, respond promptly to their needs and feedback, enhance their brand image, and build public trust and satisfaction [4-6]. Hence, social media influence is a means for expanding visibility, reputation, and critical support for achieving long-term sustainable development for hospitals [7,8].

Existing studies suggest that social media influence primarily encompasses 4 dimensions: audience reach, audience approval, audience interaction, and dissemination power [9-11]. Audience reach refers to the breadth and spread of information, reflecting the penetration and popularity of hospital content on social media platforms [12]. Audience approval indicates the audience’s acceptance of and trust in hospital-published information, highlighting the hospitals’ professional authority and brand image [13,14]. Audience interaction measures the quantity of user-hospital engagement on the platform, indicating the closeness between hospitals and the public [15]. Dissemination power focuses on content sharing and diffusion capability, assessing the impact and reach of secondary transmissions [16].

A hospital’s comprehensive capabilities are directly linked to its social media influence [17,18]. Large hospitals with stronger medical resources, professional talent, and broader patient bases enjoy inherent exposure and authority on social media [19]. These hospitals leverage their reputation and brand advantages to attract public attention and gain higher trust and recognition when sharing medical information [20].

However, a hospital’s comprehensive capabilities require long-term accumulation, which is challenging to enhance significantly in the short term. Promotional strategies are another key approach for enhancing hospitals’ social media influence [21,22]. Promotional strategies mainly focus on 3 aspects: activity, interactivity, and entertainment value. Activity emphasizes the frequency and timeliness of content updates, maintaining platform engagement through regular updates of health knowledge and medical information [23-25]. Interactivity emphasizes the frequency and quality of engagement between hospitals and other accounts, strengthening social networks through active and meaningful responses [26,27]. Entertainment value involves creating engaging and visually attractive content such as multimedia posts and short videos to enhance the appeal and dissemination of information [28,29]. With carefully curated content, effective interaction management, and collaborations with medical experts or influencers, hospitals can significantly boost their social media influence in the short term.

Although the aforementioned studies demonstrate that promotional strategies can effectively improve a hospital’s social media performance, the specific mechanisms impacting the 4 dimensions of social media influence remain underexplored. In addition, the effectiveness of promotional strategies may vary across different types of hospitals, and tailored strategies are crucial for optimizing resource allocation and enhancing promotional efficacy. Further research is needed to clarify how promotional strategies affect various dimensions of social media influence and identify heterogeneity across hospital types to provide actionable strategic recommendations.

In this study, we formulated hypotheses based on the uses and gratifications theory [30,31], dialogic communication theory [32,33], elaboration likelihood model [34,35], resource-based view [36,37], and institutional theory [38]. We empirically examined how the 3 dimensions of promotional strategies (activity, interactivity, and entertainment value) influence the 4 aspects of hospital social media influence (audience reach, audience approval, audience interaction, and dissemination power). In addition, hospital characteristics were incorporated as moderating variables to further explore their effects on these relationships.

Data were extracted from 721 verified hospital Weibo accounts and merged with various hospital characteristics sourced from the DXY National Hospital Database. Weibo, one of China’s largest social media platforms with a vast user base and robust information dissemination capabilities, has become a key venue for hospital social media promotion [39-41]. Within this context, this study collected data from hospital Weibo accounts officially verified as of January 2024.

The results of this study reveal how various promotional strategies impact the dimensions of social media influence and offer evidence-based guidance for formulating targeted strategies tailored to various hospital types. These findings not only help enhance hospital influence and public trust on social media platforms but also lay a solid foundation for future brand building and sustainable development.

### Hypotheses

#### Relationship Between Activity and Social Media Influence

According to the uses and gratifications theory [30,31], users actively select specific media to fulfill their informational, social interaction, and personal cognitive and emotional needs. The frequency and timeliness of information updates on social media determine the availability of information, subsequently affecting user satisfaction. Specifically, in the hospital context, frequent updates of professional content such as health education and medical service developments can effectively satisfy users’ demand for medical information. This can increase users’ attention to and appreciation for the hospital’s content, potentially correlating with higher user engagement and broader dissemination effects.

In addition, frequent updates can enhance content visibility on social platforms, reinforcing continuous content dissemination and increasing its reach. Consequently, it can be hypothesized that hospitals more active on social media will exhibit higher influence metrics (eg, followers, likes, comments, and shares). Thus, we propose the following hypothesis: hospital social media activity (number of posts) is positively associated with audience reach (followers), audience approval (likes), audience interaction (comments), and dissemination capability (shares; hypothesis 1).

#### Relationship Between Interactivity and Social Media Influence

Drawing from dialogic communication theory [32,33], effective communication emphasizes not merely the 1-way transfer of information but also 2-way interaction and response. For medical institutions, social media interactions (eg, proactively following other accounts and promptly responding to user comments) reflect hospitals’ willingness and transparency in communicating with the public. This interaction enhances public trust and relationship quality, encouraging users to respond more actively to hospital posts and share content further.

Moreover, hospitals that proactively engage with other relevant accounts can expand their social networks, increasing the frequency and density of network interactions, thereby enhancing information dissemination. Consequently, this study suggests that hospitals with higher interactivity levels will achieve higher social media influence. Thus, we propose the following hypothesis: hospital interactivity (number of other accounts followed) is positively associated with audience reach (followers), audience approval (likes), audience interaction (comments), and dissemination capability (shares; hypothesis 2).

#### Relationship Between Entertainment Value and Social Media Influence

According to the elaboration likelihood model [34,35], information processing can occur through either a central or peripheral route. Typically, entertainment content (such as images or videos), characterized by intuitive, vivid, and easily understandable features, serves as a peripheral cue, quickly capturing user attention and stimulating superficial interactions (likes and follows). However, when such content integrates substantial professional information, data, or specific facts, users may engage in deeper cognitive processing via the central route, leading to deeper interactions such as comments and shares. Consequently, we hypothesize that entertainment content positively impacts various dimensions of hospital social media influence. Therefore, we propose that the entertainment value of hospital social media (percentage of posts containing images and videos) is positively associated with audience reach (followers), audience approval (likes), audience interaction (comments), and dissemination capability (shares; hypothesis 3).

#### Moderating Role of Hospital Characteristics

On the basis of the resource-based view [36,37], an organization’s strategic advantage is derived from its internal resources, which must be valuable, rare, and difficult to imitate. In the context of health care institutions, larger hospitals or those with higher rankings typically possess more professional expertise, richer medical resources, and stronger brand influence. These key resources facilitate the creation of authoritative and credible professional content on social media, enhancing the breadth and depth of information dissemination. Hence, larger or higher-ranking hospitals, leveraging their resource advantages, are likely to achieve better outcomes from their social media strategies.

In addition, institutional theory [38] suggests that organizational behaviors are influenced by external institutional environments, including ownership structures and regional development levels. Specifically, private hospitals typically demonstrate stronger market orientation and operational flexibility, enabling rapid responses to market demands and adjustments in social media strategies, thus enhancing audience interaction and dissemination effectiveness. Conversely, public hospitals are often constrained by government policies and financial management practices, potentially limiting their flexibility and effectiveness on social media. Furthermore, regional economic and social development significantly affects public preferences and demands for medical information, influencing the effectiveness of hospital social media strategies. On the basis of these analyses, we propose that hospital characteristics (size, ranking, ownership, and region) significantly moderate the relationship between promotional strategies and social media influence (hypothesis 4).

## Methods

### Data

#### Weibo Dataset

This study was based on data from hospital accounts officially verified by the Chinese Weibo platform, ensuring the authenticity and reliability of the data sources. Specifically, the dataset includes comprehensive social media information for each hospital’s Weibo account up to January 2024. Key account-level metrics such as the number of followers, accounts followed, and total published posts were collected. In addition, details of each post, including its content and engagement metrics (eg, likes, comments, and shares), were obtained to reflect the operational performance of hospital Weibo accounts and audience engagement comprehensively.

#### Hospital Characteristic Dataset

To comprehensively analyze the impact of promotional strategies on hospitals’ social media influence, this study incorporated multidimensional hospital characteristic data from the DXY National Hospital Database. These characteristics include basic attributes and developmental status, such as the hospital’s location (province and city), ownership (public or private), specialization (general or specialized), grade (eg, primary, secondary, and tertiary, with tertiary being the highest level), and size (measured through the number of beds). In addition, indicators reflecting the hospital’s historical foundation and brand influence were included, such as the year of establishment, developmental history, and national brand rankings. This paper also incorporates information at the level of the province where the hospitals are located. The data were sourced from the China Statistical Yearbook [42].

#### Data Integration

The final dataset integrates social media data from the Weibo platform with hospital characteristic information from the DXY database, creating a comprehensive multidimensional dataset encompassing 721 hospital Weibo accounts. This dataset captures both the online performance of hospitals on social media and their offline comprehensive capabilities and characteristics. This integration ensured the thoroughness of the analysis and the scientific validity of this study’s conclusions. Using this dataset, this study explored the impact of hospital promotional strategies on social media influence from both online and offline dimensions.

### Variables

This study investigated how to use promotional strategies to enhance the social media influence of hospitals. Hospital social media influence was categorized into 4 dimensions: audience reach, audience approval, audience interaction, and dissemination capacity. In detail, we measured audience reach on social media using the number of followers of the hospitals’ official accounts on Weibo (*wbFans* variable). Audience approval was evaluated using the maximum number of likes received on a single Weibo post (*wbLike* variable). Audience interaction was assessed through the maximum number of comments on a Weibo post (*wbComment* variable), whereas dissemination power was reflected in the maximum number of reposts of a single Weibo post (*wbRetweet* variable).

Next, this study examined the promotional strategies used by hospitals on social media focusing on 3 key dimensions: activity, interactivity, and entertainment value. Specifically, account activity was measured using the number of posts made by the hospitals’ official accounts on Weibo (*wbNum* variable). Interactivity was represented by the number of accounts followed by the hospitals accounts (*wbFollow* variable). Entertainment value was assessed through the percentage of posts containing images (*wbPicture* variable) and videos (*wbVideo* variable) relative to the total number of posts.

Finally, recognizing the diverse characteristics of hospitals, we considered several key attributes. These included the number of approved beds (*Bed* variable) as an indicator of hospital size; years of operation (*History* variable) to reflect institutional maturity; overall ranking (*Rank* variable) to represent hospital reputation; and binary indicators for whether the hospital was tertiary (*Tertiary* variable), private (*Private* variable), or general (*General* variable). In addition, we included the hospital’s provincial location. In terms of provincial characteristics, we further considered residents’ disposable income (*Income* variable), population density (*Density* variable), and age structure (the proportion of the population aged 14-65 years, denoted as the *Age* variable).

### Descriptive Statistics

This paper presents descriptive statistics for the key variables, including the sample size, mean, SD, and minimum and maximum values. In addition, we examined the geographical distribution of hospitals to explore regional patterns in their presence.

### Multivariate Linear Regression Analysis

To investigate how hospitals’ promotional strategies affected their social media influence, this study used a multiple linear regression model, as specified in equation 1. In equation 1, the dependent variable *y* represents various dimensions of hospitals’ social media influence, including the variables *wbFans*, *wbLike*, *wbComment*, and *wbRetweet*. To assess the percentage change in *y*, a logarithmic transformation was applied to these variables.

The key independent variables in equation 1 capture hospitals’ social media promotional strategies, including the variables *wbNum*, *wbFollow*, *wbPicture*, and *wbVideo*. The number of posts (*wbNum*) and accounts followed (*wbFollow*) were logarithmically transformed and represented as *ln(wbNum)* and *ln(wbFollow)*, respectively. The proportions of posts with images (*wbPicture*) and videos (*wbVideo*) were multiplied by 100 to reflect percentages, denoted as wbPicture*100 and wbVideo*100.

In addition, equation 1 incorporates a set of control variables reflecting hospital-specific characteristics, including *Bed*, *History*, *Rank*, *Tertiary*, *Private*, and *General*. The variable *Bed* was logarithmically transformed and denoted as *ln(Bed)*. Given the regional disparities across provinces in China, equation 1 also accounts for the effect of provincial location on hospitals’ social media influence. A set of province-level dummy variables *D^k^*, which indicates whether a hospital was located in the *k*th province, was included. This approach provided a simplified yet effective means of controlling for unobserved characteristics associated with a hospital’s location. Given that provinces differ in terms of economic development, population structure, and other contextual factors that may systematically influence hospitals’ social media performance, the inclusion of *D^k^* captured the composite effects of province-level attributes and mitigated potential bias from omitted variable issues.

ln(y_i_) = α + β_1_ln(wbNum)_i_ +β_2_ln(wbFollow)_i_ +β_3_wbPicture_i_*100 + β_4_ wbVideo_i_*100 +γ_1_ln(Bed)_i_ +γ_2_History_i_ +γ_3_Rank_i_/100 +γ_4_Tertiary_i_ +γ_5_Private_i_ + γ_6_General_i_ + Σ_k_η_k_D^k^_i_ + u_i_ (1)

### Heterogeneity Analysis

The effectiveness of promotional strategies in enhancing social media influence may vary across different types of hospitals. This study further explored heterogeneity at the hospital level.

First, this study examined whether hospitals’ promotional strategies had already exhibited differentiation. To address this question, equation 2 was used. In equation 2, the dependent variable *y* represents various aspects of hospitals’ promotional strategies, including *ln(wbNum)*, *ln(wbFollow)*, wbPicture*100, and wbVideo*100. The independent variables in equation 2 capture hospital characteristics, including *ln(Bed)*, *History*, *Rank*, *Tertiary*, *Private*, *General*, and *D^k^*. In addition, *D^k^* represents dummy variables for the hospitals’ provincial location. A multiple linear regression was conducted to analyze these relationships.

y_i_ = α + β_1_ln(Bed)_i_ + β_2_History_i_ + β_3_Rank_i_/100 + β_4_Tertiary_i_ + β_5_Private_i_ + β_6_General_i_ + +Σ_k_η_k_D^k^_i_ + u_i_ (2)

Second, this study grouped hospitals according to various characteristics to examine potential heterogeneity in the effectiveness of promotional strategies on enhancing social media influence. Hospitals were categorized based on the following dimensions: (1) large versus small size (based on the median number of approved beds), (2) long versus short history (also proxied by the median number of approved beds), (3) high versus low comprehensive ranking (using the median rank as the threshold), (4) high versus low hospital grade (ie, whether the hospital was a tertiary institution), (5) public versus private ownership, (6) general versus specialized hospitals, and (7) geographical region (with the northeastern and central regions combined due to limited northeastern observations, resulting in 3 regional groups: eastern, central and northeastern, and western).

For binary classifications (eg, ownership, ranking, size, history, grade, and whether the hospital was general or specialized), a dummy variable *G* was introduced. Building on equation 1, the core explanatory variables—*ln(wbNum)*, *ln(wbFollow)*, *wbPicture100*, and *wbVideo100*—each interacted with *G*—(ln(wbNum)*G, ln(wbFollow)*G, wbPicture*100*G, and wbVideo_i_*100*G—as shown in equation 3. The coefficients of these interaction terms capture whether the effects of promotional strategies on social media influence differed by hospital type. Similarly, for the 3-category classification (ie, geographic region), 2 dummy variables, *G*_1_ and *G*_2_, were constructed. The interaction terms between the core explanatory variables and *G*_1_ and *G*_2_—ln(wbFollow)*G_1_, wbPicture*100*G_1_, wbVideo*100*G_1_, ln(wbNum)*G_2I_, ln(wbFollow)*G_2_, wbPicture*100*G_2_, and wbVideo*100*G_2_—were included in the regression, as specified in equation 4.

This analysis aimed to identify which factors moderated the effectiveness of promotional strategies and provide tailored policy recommendations for hospitals with different characteristics.

ln(y_i_) = α + β_1_ln(wbNum)_i_ +β_2_ln(wbFollow)_i_ +β_3_wbPicture_i_*100 + β_4_ wbVideo_i_*100 + γ_1_ln(Bed)i +γ_2_History_i_ +γ_3_Rank_i_/100 +γ_4_Tertiary_i_ +γ_5_Private_i_ + γ_6_General_i_ + Σ_k_η_k_D^k^_i_ + δ_1_ln(wbNum)_i_*G_I_ +δ_2_ln(wbFollow)_i_*G_I_+δ_3_wbPicture_i_*100*G_I_ + δ_4_ wbVideo_i_*100*G_I_ + u_i_ (3)

ln(y_i_) = α + β_1_ln(wbNum)_i_ +β_2_ln(wbFollow)_i_ +β_3_wbPicture_i_*100 + β_4_ wbVideo_i_*100 + γ_1_ln(Bed)_i_ +γ_2_History_i_ +γ_3_Rank_i_/100 +γ_4_Tertiary_i_ +γ_5_Private_i_ + γ_6_General_i_ + Σ_k_η_k_D^k^_i_ + δ_1_ln(wbNum)_i_*G_1_I +δ_2_ln(wbFollow)_i_*G_1_I+δ_3_wbPicture_i_*100*G_1I_ + δ_4_ wbVideo_i_*100*G_1I_ + σ_1I_n(wbNum)_i_*G_2I_ +σ_2_ln(wbFollow)_i_*G_2I_+σ_3_wbPicture_i_*100*G_2I_ + σ_4_ wbVideo_i_*100*G_2I_ + u_i_ (4)

### Robustness Checks

To demonstrate the robustness of the main results, we conducted several additional analyses. First, the baseline regression shown in equation 1 can be interpreted as a fixed-effects model at the province level. Building on this idea, we extended the specification by estimating a mixed-effects model, which incorporated observable province-level variables and a random intercept for each province. As shown in equation 5, the set of province-level dummy variables *D^k^* was replaced with observable province-level control variables (*Income*, *Density*, and *Age*) as well as a province-level random effect *ε_k_.* Second, to test the generalizability of the model, we compared empirical results across different sets of control variables. In addition, to address the multiple comparison issue arising from the inclusion of numerous predictors, we applied the Benjamini-Hochberg false discovery rate (FDR) procedure to adjust the *P* values and reduce the risk of false positive errors.

ln(y_i_) = α + β_1_ln(wbNum)_i_ +β_2_ln(wbFollow)_i_ +β_3_wbPicture_i_*100 + β_4_ wbVideo_i_*100 +γ_1_ln(Bed)_i_ +γ_2_History_i_ +γ_3_Rank_i_/100 +γ_4_Tertiary_i_ +γ_5_Private_i_ + γ_6_General_i_ +η_1_ln(Income)_i_ +η_2_ln(Density)_k_+η_3_Age_i_ + ε_i_+ u_i_ (5)

### Ethical Considerations

This study did not require new approval from the institutional ethics committee, as it did not involve any clinical interventions and was based on publicly available data.

## Results

### Descriptive Statistics

**Figure 1:**
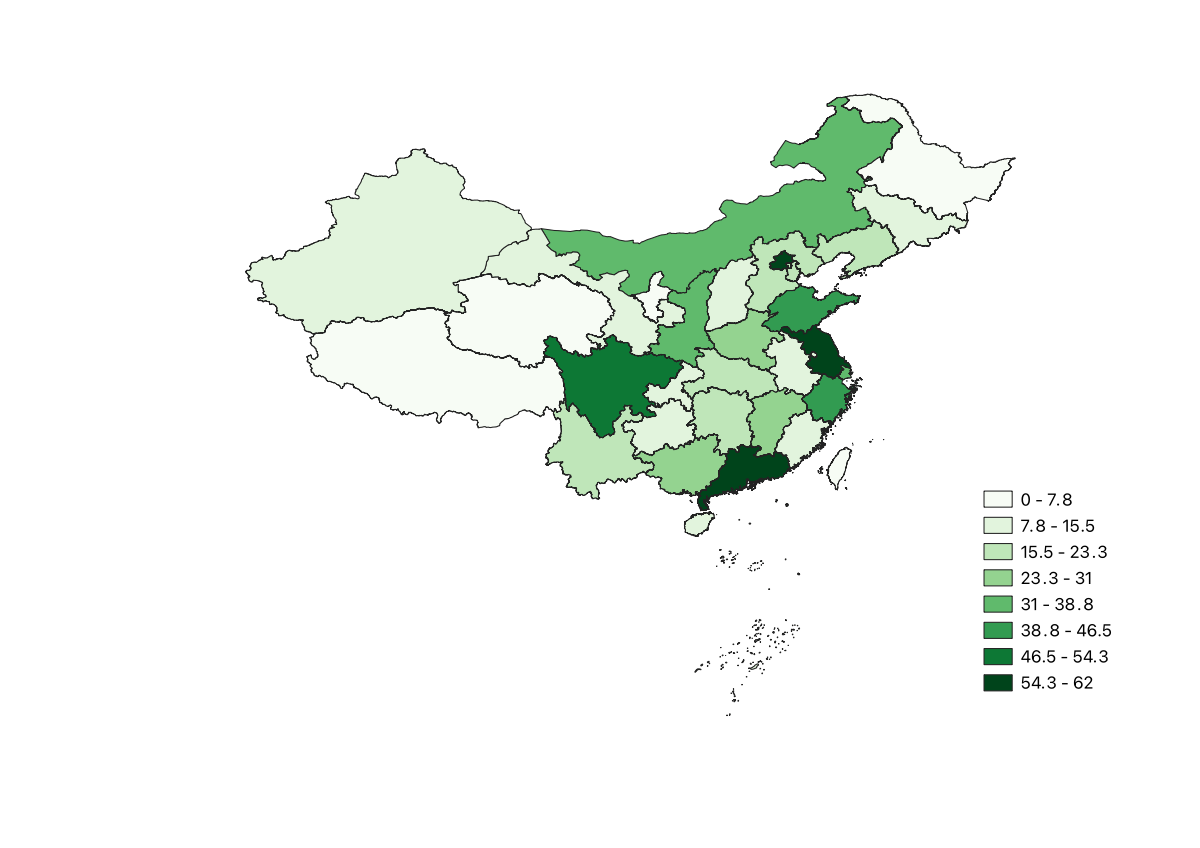
Distribution of hospitals by province.

Descriptive statistics for the variables used in this study are presented in Table 1. Furthermore, the distribution of hospitals across provinces is illustrated in Figure 1. As shown in the figure, hospital resources are unevenly distributed at the provincial level, reflecting disparities closely tied to local economic development, population size, and other regional resource conditions.

This uneven distribution across provinces provides 2 key insights. First, local resource endowments may shape the market size and social media influence of hospitals. Therefore, in analyzing hospital social media performance, it is essential to consider not only hospital-specific characteristics and promotional strategies but also regional influences. Second, Figure 1 reveals that hospitals are most densely concentrated in the eastern coastal regions, relatively sparse in the western provinces, and moderately distributed in the central and northeastern regions.

**Figure 2:**
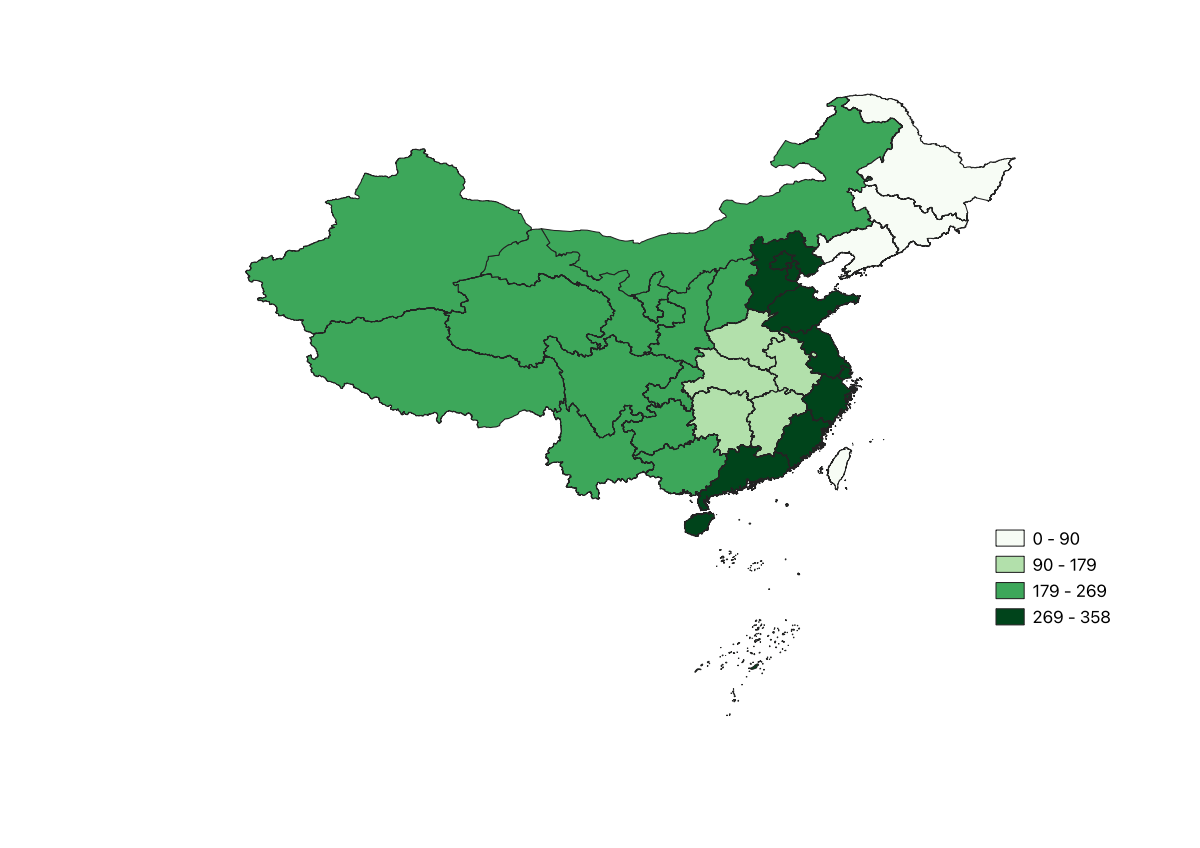
Distribution of hospitals by region.

To facilitate the analysis of regional heterogeneity, we divided China into 4 major regions: eastern (Beijing, Tianjin, Hebei, Shanghai, Jiangsu, Zhejiang, Fujian, Shandong, Guangdong, and Hainan), central (Shanxi, Anhui, Jiangxi, Henan, Hubei, and Hunan), northeastern (Liaoning, Jilin, and Heilongjiang), and western (Inner Mongolia, Guangxi, Chongqing, Sichuan, Guizhou, Yunnan, Tibet, Shaanxi, Gansu, Qinghai, Ningxia, and Xinjiang). The distribution of hospital counts across these 4 regions is presented in Figure 2.

**Table 1:** Descriptive statistics of the variables.

Variable	Sample size, n	Values, mean (SD; range)
wbFans (followers)	721	16,717.570 (80,555.360; 1-1,402,896)
wbLike (likes)	673	6266.548 (118,121.600; 0-3,017,658)
wbComment (comments)	673	837.577 (14,793.370; 0-381,010)
wbRetweet (retweets)	673	917.829 (12,154.800; 0-304,744)
Bed (beds)	583	1322.794 (917.931; 10-5936)
History (years)	671	66.921 (31.490; 7-188)
Rank (rank)	663	1491.891 (1531.056; 2-6642)
Tertiary (0=no, 1=yes)	721	0.817 (0.387; 0-1)
Public (0=private, 1=public)	721	0.906 (0.292; 0-1)
General (0=specialty, 1=general)	721	0.692 (0.462; 0-1)
wbNum (posts)	721	1377.391 (2646.232; 0-19,315)
wbFollow (followed accounts)	721	179.680 (286.094; 0-2045)
wbPicture (0=no, 1=yes)	673	0.369 (0.277; 0-1)
wbVideo (0=no, 1=yes)	673	0.087 (0.172; 0-1)

### Multivariate Linear Regression Analysis

The regression results of equation 1 are presented in Table 2. First, the activity and interactivity of hospitals’ Weibo accounts significantly influenced all 4 dimensions of social media impact. Specifically, when the dependent variables were *ln(wbFans)*, *ln(wbLike)*, *ln(wbComment)*, and *ln(wbRetweet)*, the coefficients for *ln(wbNum)* were 0.482 (95% CI 0.399-0.565; *P*<.001), 0.435 (95% CI 0.338-0.532; *P*<.001), 0.309 (95% CI 0.224-0.395; *P*<.001), and 0.489 (95% CI 0.407-0.572; *P*<.001), respectively. This indicates that a 10% increase in the number of Weibo posts by a hospital corresponded to increases of 4.8% in followers (*wbFans*), 4.4% in maximum likes (*wbLike*), 3.1% in maximum comments (*wbComment*), and 4.9% in maximum shares (*wbRetweet*). Similarly, the coefficients for *ln(wbFollow)* were 0.447 (95% CI 0.320-0.574; *P*<.001), 0.241 (95% CI 0.092-0.391; *P*=.002), 0.235 (95% CI 0.104-0.367; *P*<.001), and 0.306 (95% CI 0.179-0.432; *P*<.001) when the dependent variables were *ln(wbFans)*, *ln(wbLike)*, *ln(wbComment)*, and *ln(wbRetweet)*, respectively. This suggests that a 10% increase in the number of accounts followed by a hospital corresponds to increases of 4.5% in followers, 2.4% in maximum likes, 2.4% in maximum comments, and 3.1% in maximum shares.

Second, the entertainment value of hospitals’ Weibo accounts had a significant positive effect only on certain dimensions of social media influence, specifically audience reach and audience approval. For instance, the coefficient for wbPicture*100 was significant only when the dependent variables were *ln(wbFans)* and *ln(wbLike)*, with values of 0.006 (95% CI 0.001-0.012; *P*=.02) and 0.005 (95% CI −0.001 to 0.012; *P*=.10), respectively. This indicates that doubling the percentage of posts containing images increased followers by 0.6% and maximum likes by 0.5%. Similarly, the coefficient for wbVideo*100 was significant only for maximum likes—*ln(wbLike)*—with a value of 0.012 (95% CI 0.002-0.022; *P*=.02), indicating that doubling the percentage of posts containing videos increased maximum likes by 1.2%.

Moreover, among the hospital characteristics, hospital size and ranking had notable impacts on social media influence. Specifically, the coefficients for *ln(Bed)* were 0.466 (95% CI 0.251-0.680; *P*<.001), 0.690 (95% CI 0.438-0.942; *P*<.001), 0.381 (95% CI 0.160-0.602; *P*=.001), and 0.432 (95% CI 0.219-0.646; *P*<.001) when the dependent variables were followers, maximum likes, maximum comments, and maximum shares, respectively. This suggests that a 10% increase in the number of authorized beds was associated with increases of 4.7% in followers, 6.9% in maximum likes, 3.8% in maximum comments, and 4.3% in maximum shares. For the same dependent variables, the coefficients for *Rank/100* were −0.02 (95% CI −0.030 to −0.009; *P*<.001), −0.014 (95% CI −0.027 to −0.001; *P*=.04), −0.008 (95% CI −0.019 to 0.003; *P*=.16), and −0.011 (95% CI −0.022 to −0.000; *P*=.049), respectively. This indicates that, except for maximum likes, a 100-rank improvement in hospital ranking increased followers by 2%, maximum comments by 1.4%, and maximum shares by 1.1%.

**Table 2:** Factors affecting hospital social media influence.

	Regression coefficient (95% CI)					*P* value			
	ln(wbFans)^a^	ln(wbLike)^b^	ln(wbComment)^c^	ln(wbRetweet)^d^	ln(wbFans)		ln(wbLike)	ln(wbComment)	ln(wbRetweet)
ln(wbNum)	0.482^e^ (0.399 to 0.565)	0.435^e^ (0.338 to 0.532)	0.309^e^ (0.224 to 0.395)	0.489^e^ (0.407 to 0.572)	<.001		<.001	<.001	<.001
ln(wbFollow)	0.447^e^ (0.320 to 0.574)	0.241^e^ (0.092 to 0.391)	0.235^e^ (0.104 to 0.367)	0.306^e^ (0.179 to 0.432)	<.001		.002	<.001	<.001
wbPicture*100	0.006^f^ (0.001 to 0.012)	0.005^g^ (–0.001 to 0.012)	0.000 (–0.005 to 0.006)	0.003 (–0.002 to 0.008)	.02		.10	.89	.28
wbVideo*100	−0.000 (–0.009 to 0.008)	0.012^f^ (0.002 to 0.022)	−0.006 (–0.015 to 0.002)	−0.002 (–0.010 to 0.006)	.96		.02	.15	.61
ln(Bed)	0.466^e^ (0.251 to 0.680)	0.690^e^ (0.438 to 0.942)	0.381^e^ (0.160 to 0.602)	0.432^e^ (0.219 to 0.646)	<.001		<.001	.001	<.001
History	−0.004 (–0.010 to 0.001)	−0.002 (–0.008 to 0.004)	0.000 (–0.005 to 0.006)	0.003 (–0.003 to 0.008)	.11		.51	.88	.34
Rank/100	−0.020^e^ (−0.030 to −0.009)	−0.014^f^ (−0.027 to −0.001)	−0.008 (–0.019 to 0.003)	−0.011^f^ (−0.022 to −0.000)	<.001		.04	.16	.049
Tertiary	0.484 (–0.374 to 1.342)	0.539 (–0.471 to 1.549)	0.236 (–0.651 to 1.122)	−0.128 (–0.983 to 0.727)	.27		.30	.60	.77
Private	0.287 (–0.879 to 0.305)	0.213 (–0.909 to 0.484)	0.139 (–0.751 to 0.472)	0.142 (–0.732 to 0.448)	.34		.55	.65	.64
General	−0.423^f^ (−0.787 to −0.058)	−0.029 (–0.458 to 0.400)	−0.171 (–0.547 to 0.205)	−0.405^f^ (−0.768 to −0.042)	.02		.89	.37	.03
D^k^	Yes	Yes	Yes	Yes	Yes		Yes	Yes	Yes
Constant	−0.752 (–2.316 to 0.812)	−5.444^e^ (−7.284 to −3.603)	−2.383^e^ (−3.999 to −0.768)	−3.816^e^ (−5.374 to −2.258)	.35		<.001	.004	<.001

^a^n=530; *R*^2^=0.482; adjusted *R*^2^=0.442.

^b^n=530; *R*^2^=0.326; adjusted *R*^2^=0.274.

^c^n=530; *R*^2^=0.251; adjusted *R*^2^=0.194.

^d^n=530; *R*^2^=0.424; adjusted *R*^2^=0.379.

^e^*P*<.01.

^f^*P*<.05.

^g^*P*<.10.

### Heterogeneity Analysis

First, the regression results of equation 2 are presented in Table 3, indicating that promotional strategies did not exhibit significant differences across hospital types, with only certain characteristics such as ranking and ownership influencing some aspects of promotional strategies. Specifically, after controlling for other major hospital characteristics, higher-ranked hospitals tended to exhibit greater activity on Weibo. When the dependent variable was *ln(wbNum)*, the coefficient for *Rank/100* was −0.037 (95% CI −0.052 to −0.022; *P*<.001), indicating that, for every 100-place improvement in ranking, the volume of Weibo posts increased by 3.7%.

**Table 3:** The influence of hospital characteristics on promotional strategies.

	Estimate (95% CI)					*P* value			
	ln(wbNum)^a^	ln(wbFollow)^b^	wbPicture*100^c^	wbVideo*100^d^	ln(wbNum)		ln(wbFollow)	wbPicture*100	wbVideo*100
ln(Bed)	0.197 (–0.105 to 0.498)	0.0284 (–0.151 to 0.207)	0.122 (–3.497 to 3.742)	0.963 (–1.414 to 3.340)	.20		.76	.95	.43
History	−0.001 (–0.009 to 0.006)	−0.001 (–0.006 to 0.003)	−0.022 (–0.111 to 0.068)	−0.035 (–0.094 to 0.024)	.71		.64	.63	.25
Rank/100	−0.037^e^ (−0.052 to −0.022)	−0.003 (–0.012 to 0.006)	−0.006 (–0.186 to 0.173)	−0.002 (–0.120 to 0.116)	<.001		.55	.95	.97
Tertiary	−0.272 (–1.477 to 0.934)	0.185 (–0.530 to 0.901)	−4.389 (–18.846 to 10.068)	5.390 (–4.105 to 14.886)	.66		.61	.55	.27
Private	1.175^e^ (−2.015 to −0.335)	1.080^e^ (−1.579 to −0.581)	−0.346 (–9.501 to 10.193)	1.247 (–7.715 to 5.220)	.006		<.001	.95	.71
General	−0.150 (–0.659 to 0.359)	−0.027 (–0.329 to 0.275)	1.397 (–4.760 to 7.553)	0.333 (–3.711 to 4.376)	.56		.86	.66	.87
D^k^	Yes	Yes	Yes	Yes	Yes		Yes	Yes	Yes
Constant	6.207^e^ (4.183 to 8.231)	5.101^e^ (3.899 to 6.302)	41.16^e^ (17.174 to 65.137)	0.868 (–14.884 to 16.619)	<.001		<.001	.001	.91

^a^n=565; *R*^2^=0.065; adjusted *R*^2^=0.003.

^b^n=565; *R*^2^=0.043; adjusted *R*^2^=–0.020.

^c^n=530; *R*^2^=0.002; adjusted *R*^2^=–0.067.

^d^n=530; *R*^2^=0.007; adjusted *R*^2^=–0.061.

^e^*P*<.01.

Specifically, public or private ownership and hospital size significantly affected the role of the entertainment value of social media content in enhancing social media influence. As shown in Table 4, when the dependent variables were ln(wbFans), ln(wbLike), ln(wbComment), and ln(wbRetweet), respectively, the coefficients for wbPicture*100*Private were 0.023 (95% CI 0.003-0.044; *P*=.03), 0.022 (95% CI −0.002 to 0.047; *P*=.07), 0.029 (95% CI 0.007-0.050; *P*=.009), and 0.024 (95% CI 0.004-0.045; *P*=.02), and the coefficients for wbVideo*100*Private were 0.036 (95% CI 0.004-0.068; *P*=.03), 0.027 (95% CI −0.011 to 0.065; *P*=.17), 0.036 (95% CI 0.003-0.070; *P*=.03), and 0.027 (95% CI −0.005 to 0.060; *P*=.09), indicating that private hospitals achieved better results across all 4 social media dimensions (audience reach, audience approval, audience interaction, and dissemination) by enhancing the entertainment value compared to public hospitals. In addition, in Table 5, *G_scale* is the dummy variable indicating whether the number of beds exceeded the sample median. Compared to small-sized hospitals, large hospitals’ entertainment content had a more significant effect in improving audience approval (the coefficient for wbPicture*100*G_scale was 0.010, 95% CI −0.001 to 0.022; *P*=.08) and engagement (the coefficient for wbPicture*100*G_scale was 0.009, 95% CI −0.001 to 0.019; *P*=.09).

Moreover, private hospitals demonstrated higher activity and interactivity on Weibo than public hospitals. When the dependent variables were *ln(wbNum)* and *ln(wbFollow)*, the coefficients for *Private* were 1.175 (95% CI 0.335-2.015; *P*=.006) and 1.080 (95% CI 0.581-1.579; *P*<.001), respectively, suggesting that private hospitals had approximately twice as many Weibo posts and followed twice as many accounts as public hospitals. Other hospital characteristics did not show significant effects on promotional strategies.

Second, we examined the impact of hospital characteristics on the effectiveness of social media promotional strategies through interaction terms. The hospital characteristics included bed count (*Bed*); years of operation (*History*); overall ranking (*Rank*); and binary indicators such as whether the hospital was a tertiary institution (*Tertiary*), private hospital (*Private*), or general hospital (*General*), as well as the region where the hospital was located. The results indicate that a hospital’s *ownership* (public or private), *size*, *ranking*, and *region* were significant factors influencing the effectiveness of social media promotional strategies.

In addition, the heterogeneous analysis in terms of ranking is shown in Table 6, where *G_rank* is the dummy variable indicating whether the rank was higher than the sample median. A hospital’s ranking significantly influenced the role of interactivity in audience interaction and dissemination on social media. The interaction term analysis showed that higher-ranked hospitals significantly increased their social media influence by enhancing interactivity, especially in the dimensions of audience interaction (the coefficients for ln(wbFollow)*G_rank were 0.183, 95% CI −0.030 to 0.396 with *P*=.09 and 0.182, 95% CI −0.022 to 0.387 with *P*=.08 when the explained variables were *ln(wbComment)* and *ln(wbRetweet)*) and content dissemination (the coefficient for wbVideo*100*G_scale was 0.017, 95% CI 0.001-0.032; *P*=.04).

**Table 4:** Factors affecting hospital social media influence with Private interaction features.

	Estimate (95% CI)					*P* value			
	ln(wbFans)^a^	ln(wbLike)^b^	ln(wbComment)^c^	ln(wbRetweet)^d^	ln(wbFans)		ln(wbLike)	ln(wbComment)	ln(wbRetweet)
ln(wbNum)	0.482^e^ (0.394 to 0.569)	0.419^e^ (0.316 to 0.521)	0.283^e^ (0.193 to 0.373)	0.465^e^ (0.378 to 0.551)	<.001		<.001	<.001	<.001
ln(wbFollow)	0.447^e^ (0.313 to 0.581)	0.263^e^ (0.104 to 0.421)	0.265^e^ (0.127 to 0.402)	0.333^e^ (0.200 to 0.466)	<.001		.001	<.001	<.001
wbPicture*100	0.005^f^ (–0.001 to 0.010)	0.004 (–0.003 to 0.010)	−0.002 (–0.007 to 0.004)	0.001 (–0.004 to 0.006)	.08		.27	.53	.71
wbVideo*100	−0.003 (–0.011 to 0.006)	0.010^g^ (0.000 to 0.020)	−0.008^f^ (–0.017 to 0.000)	−0.004 (–0.012 to 0.005)	.52		.04	.06	.40
ln(Bed)	0.487^e^ (0.273 to 0.701)	0.714^e^ (0.461 to 0.966)	0.413^e^ (0.193 to 0.633)	0.459^e^ (0.247 to 0.672)	<.001		<.001	<.001	<.001
History	−0.004 (–0.009 to 0.001)	−0.002 (–0.008 to 0.004)	0.001 (–0.005 to 0.006)	0.003 (–0.002 to 0.008)	.12		.56	.78	.28
Rank/100	−0.020^e^ (−0.030 to −0.009)	−0.013^g^ (−0.026 to −0.001)	−0.007 (–0.019 to 0.004)	−0.010^f^ (–0.021 to 0.001)	<.001		.04	.19	.06
Tertiary	0.557 (–0.308 to 1.422)	0.657 (–0.364 to 1.678)	0.398 (–0.492 to 1.287)	0.021 (–0.840 to 0.881)	.21		.21	.38	.96
Public	−1.501 (–4.311 to 1.309)	−1.277 (–4.593 to 2.039)	−2.227 (–5.115 to 0.662)	−1.775 (–4.570 to 1.019)	.29		.45	.13	.21
General	−0.460^g^ (−0.824 to −0.097)	−0.056 (–0.486 to 0.373)	−0.205 (–0.579 to 0.169)	−0.431^g^ (−0.793 to −0.069)	.01		.80	.28	.02
ln(wbNum)*Private	0.026 (–0.255 to 0.308)	0.133 (–0.199 to 0.466)	0.232 (–0.057 to 0.521)	0.205 (–0.075 to 0.485)	.85		.43	.12	.15
ln(wbFollow)*Private	0.056 (–0.333 to 0.444)	−0.098 (–0.556 to 0.361)	−0.114 (–0.513 to 0.286)	−0.116 (–0.503 to 0.270)	.78		.68	.58	.56
wbPicture*100*Private	0.023^g^ (0.003 to 0.044)	0.022^f^ (–0.002 to 0.047)	0.029^e^ (0.007 to 0.050)	0.024^g^ (0.004 to 0.045)	.03		.07	.009	.02
wbVideo*100*Private	0.036^g^ (0.004 to 0.068)	0.027 (–0.011 to 0.065)	0.036^g^ (0.003 to 0.070)	0.027^f^ (–0.005 to 0.060)	.03		.17	.03	.09
D^k^	Yes	Yes	Yes	Yes	Yes		Yes	Yes	Yes
Constant	−1.161 (–2.745 to 0.423)	−5.860^e^ (−7.730 to −3.991)	−2.790^e^ (−4.418 to −1.162)	−4.198^e^ (−5.774 to −2.623)	.15		<.001	.001	<.001

^a^n=530; *R*^2^=0.491; adjusted *R*^2^=0.447.

^b^n=530; *R*^2^=0.333; adjusted *R*^2^=0.276.

^c^n=530; *R*^2^=0.271; adjusted *R*^2^=0.208.

^d^n=530; *R*^2^=0.435; adjusted *R*^2^=0.386.

^e^*P*<.01.

^f^*P*<.10.

^g^*P*<.05.

**Table 5:** Factors affecting hospital social media influence with Scale interaction features.

	Estimate (95% CI)					*P* value			
	ln(wbFans)^a^	ln(wbLike)^b^	ln(wbComment)^c^	ln(wbRetweet)^d^	ln(wbFans)		ln(wbLike)	ln(wbComment)	ln(wbRetweet)
ln(wbNum)	0.420^e^ (0.312 to 0.528)	0.388^e^ (0.260 to 0.515)	0.290^e^ (0.179 to 0.402)	0.426^e^ (0.319 to 0.532)	<.001		<.001	<.001	<.001
ln(wbFollow)	0.442^e^ (0.285 to 0.599)	0.212^f^ (0.027 to 0.398)	0.163^f^ (0.002 to 0.325)	0.277^e^ (0.122 to 0.433)	<.001		.03	.048	<.001
wbPicture*100	0.003 (–0.004 to 0.010)	0.000 (–0.008 to 0.009)	−0.003 (–0.011 to 0.004)	−0.001 (–0.008 to 0.006)	.45		.91	.34	.83
wbVideo*100	0.003 (–0.009 to 0.014)	0.012^g^ (–0.002 to 0.026)	−0.007 (–0.018 to 0.005)	−0.003 (–0.014 to 0.008)	.65		.08	.28	.62
ln(Bed)	0.143 (–0.125 to 0.410)	0.299^g^ (–0.016 to 0.614)	−0.011 (–0.286 to 0.264)	0.019 (–0.245 to 0.283)	.30		.06	.94	.89
History	−0.004^g^ (–0.010 to 0.001)	−0.002 (–0.008 to 0.004)	0.000 (–0.005 to 0.006)	0.002 (–0.003 to 0.008)	.10		.48	.94	.36
Rank/100	−0.018^e^ (−0.029 to −0.008)	−0.012^g^ (–0.024 to 0.001)	−0.006 (–0.017 to 0.005)	−0.009 (–0.019 to 0.002)	.001		.07	.31	.11
Tertiary	0.424 (–0.424 to 1.272)	0.465 (–0.533 to 1.464)	0.174 (–0.698 to 1.047)	−0.209 (–1.047 to 0.628)	.33		.36	.70	.62
Public	0.213 (–0.373 to 0.799)	0.115 (–0.575 to 0.805)	0.039 (–0.563 to 0.642)	0.040 (–0.539 to 0.619)	.48		.74	.90	.89
General	−0.471^f^ (−0.833 to −0.110)	−0.094 (–0.519 to 0.331)	−0.231 (–0.602 to 0.141)	−0.473^e^ (−0.830 to −0.116)	.01		.66	.22	.009
ln(wbNum)*G_scale	0.105 (–0.039 to 0.249)	0.072 (–0.098 to 0.241)	0.012 (–0.136 to 0.160)	0.104 (–0.039 to 0.246)	.15		.41	.87	.15
ln(wbFollow)*G_scale	−0.017 (–0.218 to 0.184)	0.032 (–0.204 to 0.269)	0.125 (–0.081 to 0.332)	0.023 (–0.175 to 0.221)	.87		.79	.24	.82
wbPicture*100*G_scale	0.008 (–0.002 to 0.017)	0.010^g^ (–0.001 to 0.022)	0.009^g^ (–0.001 to 0.019)	0.008 (–0.002 to 0.017)	.12		.08	.09	.11
wbVideo*100*G_scale	−0.007 (–0.022 to 0.009)	−0.001 (–0.019 to 0.017)	−0.001 (–0.016 to 0.015)	−0.000 (–0.015 to 0.015)	.40		.91	.94	.99
D^k^	Yes	Yes	Yes	Yes	Yes		Yes	Yes	Yes
Constant	1.377 (–0.598 to 3.353)	−2.745^f^ (−5.071 to −0.419)	0.372 (–1.659 to 2.404)	−0.865 (–2.816 to 1.086)	.17		.02	.72	.38

^a^n=530; *R*^2^=0.500; adjusted *R*^2^=0.456.

^b^n=530; *R*^2^=0.348; adjusted *R*^2^=0.292.

^c^n=530; *R*^2^=0.283; adjusted *R*^2^=0.221.

^d^n=530; *R*^2^=0.453; adjusted *R*^2^=0.406.

^e^*P*<.01.

^f^*P*<.05.

^g^*P*<.10.

**Table 6:** Factors affecting hospital social media influence with Rank interaction features.

	Estimate (95% CI)					*P* value			
	ln(wbFans)^a^	ln(wbLike)^b^	ln(wbComment)^c^	ln(wbRetweet)^d^	ln(wbFans)		ln(wbLike)	ln(wbComment)	ln(wbRetweet)
ln(wbNum)	0.502^e^ (0.385 to 0.620)	0.377^e^ (0.238 to 0.517)	0.353^e^ (0.231 to 0.476)	0.509^e^ (0.391 to 0.627)	<.001		<.001	<.001	<.001
ln(wbFollow)	0.363^e^ (0.195 to 0.532)	0.259^f^ (0.059 to 0.458)	0.136 (–0.039 to 0.311)	0.206^f^ (0.038 to 0.375)	<.001		.01	.13	.02
wbPicture*100	0.007^g^ (–0.001 to 0.015)	0.006 (–0.003 to 0.015)	0.000 (–0.008 to 0.008)	0.004 (–0.003 to 0.012)	.08		.20	.99	.27
wbVideo*100	−0.010^g^ (–0.023 to 0.002)	0.008 (–0.006 to 0.023)	−0.009 (–0.021 to 0.004)	−0.006 (–0.019 to 0.006)	.10		.28	.19	.30
ln(Bed)	0.412^e^ (0.196 to 0.628)	0.644^e^ (0.388 to 0.900)	0.345^e^ (0.121 to 0.570)	0.382^e^ (0.166 to 0.598)	<.001		<.001	.003	.001
History	−0.005^g^ (–0.010 to 0.001)	−0.002 (–0.008 to 0.004)	0.000 (–0.005 to 0.006)	0.002 (–0.003 to 0.008)	.09		.52	.97	.40
Rank/100	−0.005 (–0.020 to 0.010)	−0.001 (–0.019 to 0.016)	0.001 (–0.014 to 0.017)	0.002 (–0.013 to 0.017)	.53		.88	.87	.78
Tertiary	0.362 (–0.498 to 1.222)	0.403 (–0.615 to 1.421)	0.159 (–0.735 to 1.053)	−0.238 (–1.097 to 0.621)	.41		.44	.73	.59
Public	0.342 (–0.248 to 0.932)	0.220 (–0.479 to 0.918)	0.193 (–0.420 to 0.806)	0.197 (–0.392 to 0.787)	.26		.54	.54	.51
General	−0.374^f^ (−0.738 to −0.011)	−0.004 (–0.434 to 0.427)	−0.138 (–0.516 to 0.239)	−0.358^g^ (–0.721 to 0.005)	.04		.99	.47	.05
ln(wbNum)*G_rank	−0.036 (–0.185 to 0.113)	0.102 (–0.074 to 0.278)	−0.083 (–0.237 to 0.072)	−0.043 (–0.192 to 0.106)	.63		.26	.29	.57
ln(wbFollow)*G_rank	0.151 (–0.053 to 0.356)	−0.031 (–0.273 to 0.211)	0.183^g^ (–0.030 to 0.396)	0.182^g^ (–0.022 to 0.387)	.15		.80	.09	.08
wbPicture*100*G_rank	−0.001 (–0.011 to 0.009)	−0.000 (–0.012 to 0.011)	0.000 (–0.010 to 0.011)	−0.002 (–0.012 to 0.007)	.89		.96	.95	.62
wbVideo*100*G_rank	0.017^f^ (0.001 to 0.032)	0.007 (–0.012 to 0.025)	0.003 (–0.013 to 0.019)	0.007 (–0.009 to 0.022)	.04		.48	.70	.41
D^k^	Yes	Yes	Yes	Yes	Yes		Yes	Yes	Yes
Constant	−0.759 (–2.340 to 0.822)	−5.420^e^ (−7.292 to −3.548)	−2.303^e^ (−3.947 to −0.660)	−3.666^e^ (−5.246 to −2.086)	.35		<.001	.006	<.001

^a^n=530; *R*^2^=0.494; adjusted *R*^2^=0.450.

^b^n=530; *R*^2^=0.333; adjusted *R*^2^=0.275.

^c^n=530; *R*^2^=0.259; adjusted *R*^2^=0.195.

^d^n=530; *R*^2^=0.433; adjusted *R*^2^=0.384.

^e^*P*<.01.

^f^*P*<.05.

^g^*P*<.10.

For the regional interaction analysis, the regression results are shown in Table 7, with *G_region1* as the dummy variable indicating whether the hospital was located in the central and northeast regions and *G_region2* as the dummy variable indicating whether the hospital was located in the eastern region. Improving activity in the eastern region significantly enhanced audience interaction and dissemination. In the western and central regions, increasing the entertainment value of social media content significantly boosted social media influence, particularly regarding audience approval (the coefficient for wbVideo*100*G_region2 was 0.021, 95% CI −0.002 to 0.044; *P*=.07) and audience interaction (the coefficient for wbVideo*100*G_region2 was 0.024, 95% CI 0.003-0.044; *P*=.02).

**Table 7:** Factors affecting hospital social media influence with Regions interaction features.

	Estimate (95% CI)					*P* value			
	ln(wbFans)^a^	ln(wbLike)^b^	ln(wbComment)^c^	ln(wbRetweet)^d^	ln(wbFans)		ln(wbLike)	ln(wbComment)	ln(wbRetweet)
ln(wbNum)	0.507^e^ (0.392 to 0.621)	0.508^e^ (0.374 to 0.641)	0.334^e^ (0.217 to 0.452)	0.552^e^ (0.440 to 0.664)	<.001		<.001	<.001	<.001
ln(wbFollow)	0.446^e^ (0.261 to 0.631)	0.135 (–0.080 to 0.350)	0.148 (–0.041 to 0.337)	0.173^f^ (–0.007 to 0.354)	<.001		.22	.12	.06
wbPicture*100	0.010^e^ (0.003 to 0.018)	0.004 (–0.005 to 0.013)	−0.002 (–0.010 to 0.005)	0.005 (–0.002 to 0.012)	.006		.38	.57	.18
wbVideo*100	0.003 (–0.009 to 0.015)	−0.001 (–0.015 to 0.013)	−0.018^e^ (−0.030 to −0.006)	−0.010 (–0.021 to 0.002)	.66		.86	.004	.10
ln(Bed)	0.469^e^ (0.252 to 0.686)	0.659^e^ (0.406 to 0.912)	0.353^e^ (0.131 to 0.575)	0.414^e^ (0.202 to 0.627)	<.001		<.001	.002	<.001
History	−0.004 (–0.010 to 0.001)	−0.002 (–0.008 to 0.004)	0.000 (–0.005 to 0.006)	0.003 (–0.003 to 0.008)	.11		.53	.87	.34
Rank/100	−0.020^e^ (−0.031 to −0.009)	−0.014^g^ (−0.027 to −0.001)	−0.008 (–0.019 to 0.004)	−0.011^f^ (–0.022 to 0.000)	<.001		.03	.19	.05
Tertiary	0.467 (–0.400 to 1.335)	0.684 (–0.326 to 1.693)	0.332 (–0.556 to 1.220)	−0.057 (–0.906 to 0.791)	.29		.18	.46	.89
Public	0.287 (–0.312 to 0.886)	0.187 (–0.510 to 0.884)	0.105 (–0.508 to 0.718)	0.086 (–0.500 to 0.672)	.35		.60	.74	.77
General	−0.427^g^ (−0.797 to −0.058)	0.018 (–0.412 to 0.448)	−0.127 (–0.506 to 0.251)	−0.390^g^ (−0.751 to −0.028)	.02		.93	.51	.04
ln(wbNum)*G_region1	−0.058 (–0.265 to 0.149)	−0.023 (–0.264 to 0.218)	0.071 (–0.141 to 0.283)	0.019 (–0.184 to 0.221)	.58		.85	.51	.86
ln(wbFollow)*G_region1	0.117 (–0.186 to 0.420)	0.226 (–0.127 to 0.579)	0.110 (–0.200 to 0.421)	0.245 (–0.052 to 0.541)	.45		.21	.49	.11
wbPicture*100*G_region1	−0.009 (–0.023 to 0.006)	−0.007 (–0.024 to 0.010)	−0.003 (–0.018 to 0.012)	−0.021^e^ (−0.035 to −0.007)	.25		.40	.65	.004
wbVideo*100*G_region1	−0.005 (–0.026 to 0.016)	0.030^g^ (0.006 to 0.055)	0.022^g^ (0.001 to 0.044)	0.017^f^ (–0.003 to 0.038)	.62		.01	.04	.09
ln(wbNum)*G_region2	−0.030 (–0.228 to 0.168)	−0.226^f^ (–0.457 to 0.004)	−0.135 (–0.337 to 0.068)	−0.196^g^ (−0.390 to −0.002)	.77		.05	.19	.047
ln(wbFollow)*G_region2	−0.105 (–0.410 to 0.201)	0.202 (–0.154 to 0.557)	0.225 (–0.087 to 0.538)	0.259^f^ (–0.040 to 0.558)	.50		.27	.16	.09
wbPicture*100*G_region2	−0.008 (–0.021 to 0.004)	0.007 (–0.008 to 0.021)	0.009 (–0.004 to 0.021)	0.004 (–0.008 to 0.016)	.19		.37	.19	.54
wbVideo*100*G_region2	−0.007 (–0.027 to 0.013)	0.021^f^ (–0.002 to 0.044)	0.024^g^ (0.003 to 0.044)	0.013 (–0.006 to 0.033)	.49		.07	.02	.19
D^k^	Yes	Yes	Yes	Yes	Yes		Yes	Yes	Yes
Constant	−1.044 (–2.638 to 0.550)	−5.612^e^ (−7.466 to −3.758)	−2.448^e^ (−4.079 to −0.816)	−3.916^e^ (−5.475 to −2.357)	.20		<.001	.003	<.001

^a^n=530; *R*^2^=0.487; adjusted *R*^2^=0.438.

^b^n=530; *R*^2^=0.347; adjusted *R*^2^=0.285.

^c^n=530; *R*^2^=0.271; adjusted *R*^2^=0.202.

^d^n=530; *R*^2^=0.450; adjusted *R*^2^=0.397.

^e^*P*<.01.

^f^*P*<.10.

^g^*P*<.05.

### Robustness Checks

This study further conducted comparative analyses of the effects of hospital promotional strategies on social media influence under different model specifications, as presented in Multimedia Appendix 1. Due to space constraints, we report only the regression coefficients and the corresponding *P* values. Models 1 to 4 refer to fixed-effects models that included only promotional strategies. Models 5 to 8 were based on the baseline specification (equation 1) with hospital-level control variables, whereas models 9 to 12 were derived from the mixed-effects model described in equation 5.

Several key findings emerge from the regression results. First, a comparison between models 5 to 8 and models 9 to 12 shows that the main findings remained consistent regardless of whether a fixed-effects or mixed-effects model was used. Second, comparing models 5 to 8 with models 1 to 4 reveals that the estimated effects were not sensitive to the inclusion or exclusion of hospital-level or provincial-level control variables. Together, these results underscore the robustness of our findings regarding the effects of hospital promotional strategies on their social media influence. Third, the mixed-effects model allowed us to examine the additional effects of provincial-level characteristics. Among these, residents’ disposable income showed a significant positive association with hospitals’ social media influence, whereas population density and age structure appeared to have no statistically significant impact.

In addition, we adjusted the *P* values using the Benjamini-Hochberg FDR procedure. Due to space limitations, we report only the original and adjusted *P* values from the baseline specification under both the fixed-effects and mixed-effects models, as shown in Multimedia Appendix 1. The results indicate that, even after the FDR adjustment, the significance levels of the key explanatory variables—hospital promotional strategies—remained stable, providing further support for the robustness of our findings.

## Discussion

### Principal Findings

This study empirically examined the relationships among 3 dimensions of hospital promotional strategies (activity, interactivity, and entertainment value) and 4 aspects of hospital social media influence (audience reach, audience approval, audience interaction, and dissemination capability) while also analyzing the moderating effects of hospital characteristics. Overall, the findings largely support the hypotheses derived from the uses and gratifications theory, dialogic communication theory, elaboration likelihood model, resource-based view, and institutional theory.

First, hospital social media activity (posting frequency) showed significant positive correlations across all dimensions of social media influence, validating hypothesis 1. According to the uses and gratifications theory, users actively seek information, social interactions, and timely updates. Hospitals that frequently post content tend to effectively fulfill these user needs and, therefore, are more likely to achieve enhanced audience reach, approval, and interaction and content dissemination. In addition, frequent, high-quality content can boost user trust and loyalty, further enhancing the hospital’s online influence [43,44].

Second, hospital social media interactivity (number of accounts followed) exhibited significant positive correlations with all aspects of social media influence, supporting hypothesis 2 and dialogic communication theory. This theory underscores the importance of 2-way communication and interactive mechanisms for building trust and deepening audience engagement. Through actively interacting with other accounts, hospitals demonstrate transparency and openness, which tends to foster increased audience responsiveness and content dissemination [45-47].

In contrast, entertainment content (such as images and videos) primarily showed significant positive correlations with audience reach and approval but not with deeper interactions and dissemination effects, partially supporting hypothesis 3. According to the elaboration likelihood model, entertainment content acts as a peripheral cue, typically prompting superficial interactions such as likes or follows. However, when this content integrates substantial professional information, it might encourage deeper cognitive engagement (comments and shares). The current scarcity of high-information content posted by hospitals likely limits the deeper dissemination potential of entertainment-oriented posts [48]. Future research should further clarify and validate distinctions between central and peripheral processing pathways.

Interaction term analyses further explored how hospital characteristics moderated promotional strategy effectiveness, offering detailed insights for tailored strategies. The examined characteristics included approved bed numbers; years of operation; overall ranking; indicators for tertiary-level, public, or general hospitals; and regional classification (eastern, northeastern, central, and western China). The results revealed significant moderating effects of ownership (public vs private), size, ranking, and region, supporting hypothesis 4.

### Ownership and Scale

Hospital ownership and size critically moderated the relationship between entertainment content and social media influence. Private hospitals outperformed others in leveraging entertainment content to boost influence, likely reflecting their greater organizational flexibility and market orientation toward user-centric content strategies. In addition, private hospitals typically prioritize public communication and brand investment, making their entertainment content more engaging. Similarly, larger hospitals were more effective than smaller ones in enhancing audience approval and interaction through entertainment content due to stronger content creation capabilities and brand reputation. According to the resource-based view, larger and private hospitals are better positioned to use internal resources to create appealing content and amplify its dissemination.

### Hospital Ranking

Hospital ranking significantly moderated the impact of interactivity on social media effectiveness. Higher-ranked hospitals effectively leveraged interactive behaviors (eg, mutual following and comments) to enhance audience interaction and dissemination capabilities, consistent with dialogic communication theory, suggesting that trusted institutions more effectively stimulate persuasive interactions, enhancing user responsiveness.

### Regional Differences

Institutional theory suggests that regional economic and social development significantly impact the effectiveness of social media promotional strategies. In the economically developed eastern region, higher activity (frequent posting) significantly improved audience interaction and dissemination, likely due to user preferences for frequent updates and algorithm-driven content visibility. In contrast, in the less economically developed central, western, and northeastern regions, increasing entertainment content more effectively enhanced audience engagement and approval, indicating that users in these areas responded more strongly to visually and emotionally engaging content. Thus, hospitals should tailor content strategies based on regional cultural and informational preferences to enhance user engagement.

In summary, hospitals currently show minimal differentiation in promotional strategies; however, future communication plans should avoid a *one-size-fits-all* approach and, instead, consider internal resources and external environmental factors. Hospitals should maintain high levels of activity and interactivity while flexibly adjusting entertainment content according to ownership, size, ranking, and regional characteristics to achieve multidimensional influence enhancement. This optimization strategy aligns with the resource-based view, emphasizing internal capabilities as strategic success factors, and resonates with institutional theory’s emphasis on external norms, regulations, and expectations, jointly guiding hospitals toward building influential and trustworthy brands in the digital era.

### Contribution

The contributions of this study are as follows. First, this study integrated multidisciplinary perspectives from communication studies (uses and gratifications theory and dialogic communication theory), marketing (elaboration likelihood model), and organizational management (resource-based view and institutional theory) to systematically analyze the relationship between hospitals’ social media promotional strategies and their online influence. This integrated analytical framework transcends the limitations of previous research, which either focused solely on internal hospital characteristics (such as size, type, and geographic location) [1,49,50] or relied primarily on small-sample case studies for preliminary evaluation of promotional effectiveness [1,4,51]. Thus, this framework provides a novel theoretical perspective for comprehensively understanding the social media communication mechanisms of professional service institutions.

Second, this study innovatively categorized hospitals’ social media promotional strategies into 3 core dimensions—activity (posting frequency), interactivity (mutual following), and entertainment value (multimedia content)—and used empirical research methods to validate the differential effects of each dimension on the 4 elements of influence (reach, approval, interaction, and dissemination power). This theoretical innovation addresses the limitations of existing literature that analyzed single functions of social media (eg, health education or brand promotion) [5,52-55], providing a more comprehensive and systematic analytical framework for health communication research.

Third, this study innovatively incorporated hospital heterogeneity (size, level, and ownership) and regional differences into the analytical framework, revealing that organizational resource endowments and institutional environments significantly moderated the effects of promotional strategies. This finding not only provides an important theoretical basis for the contextual adaptation of social media promotional strategies but also expands the application boundaries of strategic communication theory in professional service fields.

Finally, based on the empirical findings, this study proposes a strategy principle emphasizing *professional content first, supplemented by entertainment* along with differentiated implementation plans, providing scientific evidence for medical institutions to optimize social media investments under resource constraints. In addition, this principle establishes an analytical perspective that integrates theoretical insights with practical applicability. It not only applies to the health care sector but also offers valuable implications for social media marketing practices in other professional domains such as education and public services, thus enhancing both the theoretical contribution and practical significance of the research findings.

### Limitations

This study has several limitations that warrant further exploration. First, the data primarily came from the Weibo platform. While Weibo is one of the largest social media platforms in China with a broad user base and robust information dissemination capabilities, integrating data from other platforms such as WeChat Channels or Douyin can provide a more comprehensive view of hospitals’ overall social media promotional strategies. Second, this study focused on official verified accounts, ensuring data reliability and credibility. However, the potential influence of unofficial accounts on public perceptions also merits further investigation. Third, this research was based on cross-sectional data, which limits the ability to analyze dynamic changes over time. Incorporating longitudinal data in future studies could help uncover trends in hospital social media performance and promotional strategies over time.

Moreover, future research could adopt longitudinal designs or experimental methods to rigorously validate the mapping between specific promotional content and theoretical constructs. For instance, content analysis could examine how different types of social media posts (eg, information-intensive vs entertainment-oriented content) differentially activate central or peripheral route processing. In addition, experimental designs could more strictly test causal mechanisms underlying the influence of interactivity and entertainment strategies on user behavior. Furthermore, the validity of theoretical constructs could be statistically assessed through scale development and validation methods such as exploratory factor analysis and structural equation modeling, further enhancing the robustness and theoretical validity of this study’s findings.

These extensions and deeper analyses will further enrich our understanding of the mechanisms behind hospitals’ social media operations.

### Conclusions

This study provides an in-depth analysis of the relationships between 3 dimensions of hospitals’ social media promotional strategies (activity, interactivity, and entertainment value) and 4 dimensions of social media influence (audience reach, audience approval, audience interaction, and dissemination power). The findings reveal that activity and interactivity exhibit significant and broad positive associations across all dimensions, highlighting the critical importance of consistently publishing high-quality content and actively expanding interactive networks to solidify hospitals’ foundational social media influence. Although the overall contribution of *entertainment value* was relatively weaker, it can still play a supplementary role under specific contexts and for certain hospital types. The interaction term analysis revealed the alignment among hospital types, regional characteristics, and strategy elements, providing a clear road map for designing and implementing tailored strategies. The results indicate that *hospital ownership* (public vs private), *size*, *ranking*, and *regional location* are key factors influencing the effectiveness of social media promotional strategies. In summary, this study offers empirical evidence and strategic insights for health care institutions to construct more credible and patient-centered brand images amidst digital transformation, laying the groundwork for sustainable success in the digital age.

## Appendix

Multimedia Appendix 1 Supplementary tables.

## Abbreviations

FDR: false discovery rate
