# Supplementary material for: Leveraging Promotional Strategies to Enhance Hospital Influence on Social Media: Cross-Sectional Study
Source: J Med Internet Res. 2025 Aug 22;27:e70676. doi: 10.2196/70676 (PMC12413568; doi:10.2196/70676)
Supplement: Multimedia Appendix 1 [file jmir_v27i1e70676_app1.docx]

**Multimedia Appendix**

**Table 1.** Comparison of different model specifications—factors affecting hospital social media influence.

|  | Model 1—ln(wbFans)^a^ | | Model 2—ln(wbLike)^b^ | | Model 3—ln(wbComment)^c^ | | Model 4—ln(wbRetweet)^d^ | | Model 5—ln(wbFans)^e^ | | Model 6—ln(wbLike)^f^ | | Model 7—ln(wbComment)^g^ | | Model 8—ln(wbRetweet)^h^ | | Model 9—ln(wbFans)^i^ | | Model 10—ln(wbLike)^j^ | | Model 11—ln(wbComment)^k^ | | Model 12—ln(wbRetweet)^l^ | |
| --- | --- | --- | --- | --- | --- | --- | --- | --- | --- | --- | --- | --- | --- | --- | --- | --- | --- | --- | --- | --- | --- | --- | --- | --- |
|  | Regression coefficient | *P* value | Regression coefficient | *P* value | Regression coefficient | *P* value | Regression coefficient | *P* value | Regression coefficient | *P* value | Regression coefficient | *P* value | Regression coefficient | *P* value | Regression coefficient | *P* value | Regression coefficient | *P* value | Regression coefficient | *P* value | Regression coefficient | *P* value | Regression coefficient | *P* value |
|  | | | | | | | | | | | | | | | | | | | | | | | | |
| ln(wbNum) | 0.530^m^ | <.001 | 0.490^m^ | <.001 | 0.357^m^ | <.001 | 0.528^m^ | <.001 | 0.482^m^ | <.001 | 0.435^m^ | <.001 | 0.309^m^ | <.001 | 0.489^m^ | <.001 | 0.482^m^ | <.001 | 0.442^m^ | <.001 | 0.308^m^ | <.001 | 0.486^m^ | <.001 |
| ln(wbFollow) | 0.481^m^ | <.001 | 0.177^m^ | .008 | 0.176^m^ | .002 | 0.236^m^ | <.001 | 0.447^m^ | <.001 | 0.241^m^ | .002 | 0.235^m^ | <.001 | 0.306^m^ | <.001 | 0.433^m^ | <.001 | 0.245^m^ | .001 | 0.256^m^ | <.001 | 0.304^m^ | <.001 |
| wbPicture*100 | 0.006^n^ | .02 | 0.004 | .14 | −0.001 | .74 | 0.001 | .68 | 0.006^n^ | .02 | 0.005^*^ | .10 | <0.001 | .89 | 0.003 | .28 | 0.006^n^ | .03 | 0.005 | .10 | <0.001 | .87 | 0.002 | .34 |
| wbVideo*100 | 0.004 | .38 | 0.016^m^ | .001 | −0.004 | .33 | −0.001 | .77 | –<0.001 | .96 | 0.012^n^ | .02 | −0.006 | .15 | −0.002 | .61 | <0.001 | .99 | 0.013^m^ | .005 | −0.005 | .21 | −0.002 | .65 |
| ln(Bed) | —^o^ | — | — | — | — | — | — | — | 0.466^m^ | <.001 | 0.690^m^ | <.001 | 0.381^m^ | .001 | 0.432^m^ | <.001 | 0.488^m^ | <.001 | 0.723^m^ | <.001 | 0.440^m^ | <.001 | 0.483^m^ | <.001 |
| History | — | — | — | — | — | — | — | — | −0.004 | .11 | −0.002 | .51 | <0.001 | .88 | 0.003 | .34 | −0.004 | .11 | −0.001 | .74 | 0.001 | .61 | 0.003 | .23 |
| Rank/100 | — | — | — | — | — | — | — | — | −0.020^m^ | <.001 | −0.014^n^ | .04 | −0.008 | .16 | −0.011^n^ | .049 | −0.020^m^ | <.001 | −0.013^n^ | .04 | −0.007 | .18 | −0.010^p^ | .06 |
| Tertiary | — | — | — | — | — | — | — | — | 0.484 | .27 | 0.539 | .30 | 0.236 | .60 | −0.128 | .77 | 0.366 | .39 | 0.426 | .39 | 0.110 | .80 | −0.224 | .60 |
| Private | — | — | — | — | — | — | — | — | 0.287 | .34 | 0.213 | .55 | 0.139 | .65 | 0.142 | .64 | 0.358 | .22 | 0.330 | .33 | 0.256 | .38 | 0.242 | .40 |
| General | — | — | — | — | — | — | — | — | −0.423^n^ | .02 | −0.029 | .89 | −0.171 | .37 | −0.405^n^ | .03 | −0.440^n^ | .02 | −0.091 | .67 | −0.248 | .18 | −0.456^n^ | .01 |
| ln(Income) | — | — | — | — | — | — | — | — | — | — | — | — | — | — | — | — | 1.944^m^ | .007 | 1.406^p^ | .08 | 0.081 | .89 | 1.329^p^ | .09 |
| ln(density) | — | — | — | — | — | — | — | — | — | — | — | — | — | — | — | — | −0.271 | .12 | −0.284 | .15 | −0.021 | .89 | −0.303 | .11 |
| Age | — | — | — | — | — | — | — | — | — | — | — | — | — | — | — | — | −4.653 | .49 | −6.707 | .36 | 1.716 | .74 | −4.474 | .54 |
| D^k^ | √ | √ | √ | √ | √ | √ | √ | √ | √ | √ | √ | √ | √ | √ | √ | — | — | — | — | — | — | — | — | — |
| Random effect ε_k_ | — | — | — | — | — | — | — | — | — | — | — | — | — | — | — | — | √ | √ | √ | √ | √ | √ | √ | √ |
| Constant | 1.321^m^ | <.001 | −0.844^m^ | .01 | 0.045 | .87 | −1.225^m^ | <.001 | −1.039 | .20 | −5.656^m^ | <.001 | −2.523^m^ | .002 | −3.958^m^ | <.001 | −16.206^m^ | <.001 | −13.862^m^ | .004 | −4.810 | .16 | −12.751^m^ | .007 |

^a^n=673; *R*^2^=0.430; adjusted *R*^2^=0.400.

^b^n=673; *R*^2^=0.245; adjusted *R*^2^=0.207.

^c^n=673; *R*^2^=0.216; adjusted *R*^2^=0.176.

^d^n=673; *R*^2^=0.381; adjusted *R*^2^=0.349.

^e^n=530; *R*^2^=0.482; adjusted *R*^2^=0.442.

^f^n=530; *R*^2^=0.326; adjusted *R*^2^=0.274.

^g^n=530; *R*^2^=0.251; adjusted *R*^2^=0.194.

^h^n=530; *R*^2^=0.424; adjusted *R*^2^=0.379.

^i^n=530.

^j^n=530.

^k^n=530.

^l^n=530.

^m^*P*<.01.

^n^*P*<.05.

^o^

^p^*P*<.10.

**Table 2.** Benjamini-Hochberg false discovery rate adjustment of *P* values—fixed-effects model.

|  | Estimate (95% CI) | | | | *P* value | | | | Adjusted *P* value | | | |
| --- | --- | --- | --- | --- | --- | --- | --- | --- | --- | --- | --- | --- |
|  | ln(wbFans)^a^ | ln(wbLike)^b^ | ln(wbComment)^c^ | ln(wbRetweet)^d^ | ln(wbFans) | ln(wbLike) | ln(wbComment) | ln(wbRetweet) | ln(wbFans) | ln(wbLike) | ln(wbComment) | ln(wbRetweet) |
|  | | | | | | | | | | | | |
| ln(wbNum) | 0.482^e^ (0.399 to 0.565) | 0.435^e^ (0.338 to 0.532) | 0.309^e^ (0.224 to 0.395) | 0.489^e^ (0.407 to 0.572) | <.001 | <.001 | <.001 | <.001 | <.001 | <.001 | <.001 | <.001 |
| ln(wbFollow) | 0.447^e^ (0.320 to 0.574) | 0.241^e^ (0.092 to 0.391) | 0.235^e^ (0.104 to 0.367) | 0.306^e^ (0.179 to 0.432) | <.001 | .002 | <.001 | <.001 | <.001 | .004 | .003 | <.001 |
| wbPicture*100 | 0.006^f^ (0.001 to 0.012) | 0.005^g^ (–0.001 to 0.012) | 0.000 (–0.005 to 0.006) | 0.003 (–0.002 to 0.008) | .02 | .10 | .89 | .28 | .04 | .15 | .89 | .43 |
| wbVideo*100 | −0.000 (–0.009 to 0.008) | 0.012^f^ (0.002 to 0.022) | −0.006 (–0.015 to 0.002) | −0.002 (–0.010 to 0.006) | .96 | .02 | .15 | .61 | .96 | .03 | .34 | .75 |
| ln(Bed) | 0.466^e^ (0.251 to 0.680) | 0.690^e^ (0.438 to 0.942) | 0.381^e^ (0.160 to 0.602) | 0.432^e^ (0.219 to 0.646) | <.001 | <.001 | .001 | <.001 | <.001 | <.001 | .003 | <.001 |
| History | −0.004 (–0.010 to 0.001) | −0.002 (–0.008 to 0.004) | 0.000 (–0.005 to 0.006) | 0.003 (–0.003 to 0.008) | .11 | .51 | .88 | .34 | .17 | .62 | .97 | .47 |
| Rank/100 | −0.020^e^ (−0.030 to −0.009) | −0.014^f^ (−0.027 to −0.001) | −0.008 (–0.019 to 0.003) | −0.011^f^ (−0.022 to −0.000) | <.001 | .04 | .16 | .049 | .001 | .07 | .28 | .09 |
| Tertiary | 0.484 (–0.374 to 1.342) | 0.539 (–0.471 to 1.549) | 0.236 (–0.651 to 1.122) | −0.128 (–0.983 to 0.727) | .27 | .30 | .60 | .77 | .33 | .41 | .83 | .77 |
| Private | 0.287 (–0.305 to 0.879) | 0.213 (–0.484 to 0.909) | 0.139 (–0.472 to 0.751) | 0.142 (–0.448 to 0.732) | .34 | .55 | .65 | .64 | .38 | .60 | .80 | .70 |
| General | −0.423^f^ (−0.787 to −0.058) | −0.029 (–0.458 to 0.400) | −0.171 (–0.547 to 0.205) | −0.405^f^ (−0.768 to −0.042) | .02 | .89 | .37 | .03 | .04 | .89 | .59 | .06 |
| D^k^ | √ | √ | √ | √ | √ | √ | √ | √ | √ | √ | √ | √ |
| Constant | −1.039 (–2.615 to 0.537) | −5.656^e^ (−7.511 to −3.802) | −2.523^e^ (−4.150 to −0.895) | −3.958^e^ (−5.528 to −2.389) | .20 | <.001 | .002 | <.001 | .27 | <.001 | .007 | <.001 |

^a^n=530; *R*^2^=0.482; adjusted *R*^2^=0.442.

^b^n=530; *R*^2^=0.326; adjusted *R*^2^=0.274.

^c^n=530; *R*^2^=0.251; adjusted *R*^2^=0.194.

^d^n=530; *R*^2^=0.424; adjusted *R*^2^=0.379.

^e^*P*<.01.

^f^*P*<.05.

^g^*P*<.10.

**Table 3.** Benjamini-Hochberg false discovery rate adjustment of *P* values—mixed-effects model.

|  | Estimate (95% CI) | | | | *P* value | | | | Adjusted *P* value | | | |
| --- | --- | --- | --- | --- | --- | --- | --- | --- | --- | --- | --- | --- |
|  | ln(wbFans)^a^ | ln(wbLike)^b^ | ln(wbComment)^c^ | ln(wbRetweet)^d^ | ln(wbFans) | ln(wbLike) | ln(wbComment) | ln(wbRetweet) | ln(wbFans) | ln(wbLike) | ln(wbComment) | ln(wbRetweet) |
|  | | | | | | | | | | | | |
| ln(wbNum) | 0.482^e^ (0.404 to 0.561) | 0.442^e^ (0.350 to 0.534) | 0.308^e^ (0.229 to 0.387) | 0.486^e^ (0.408 to 0.565) | <.001 | <.001 | <.001 | <.001 | <.001 | <.001 | <.001 | <.001 |
| ln(wbFollow) | 0.433^e^ (0.311 to 0.554) | 0.245^e^ (0.103 to 0.387) | 0.256^e^ (0.133 to 0.379) | 0.304^e^ (0.182 to 0.425) | <.001 | .001 | <.001 | <.001 | <.001 | .003 | <.001 | <.001 |
| wbPicture*100 | 0.006^f^ (0.001 to 0.011) | 0.005 (–0.001 to 0.011) | 0.000 (–0.005 to 0.006) | 0.002 (–0.003 to 0.008) | .03 | .10 | .87 | .34 | .049 | .16 | .99 | .46 |
| wbVideo*100 | 0.000 (–0.008 to 0.008) | 0.013^e^ (0.004 to 0.023) | −0.005 (–0.013 to 0.003) | −0.002 (–0.010 to 0.006) | .99 | .005 | .21 | .65 | .99 | .01 | .38 | .65 |
| ln(Bed) | 0.488^e^ (0.281 to 0.695) | 0.723^e^ (0.481 to 0.965) | 0.440^e^ (0.229 to 0.650) | 0.483^e^ (0.276 to 0.690) | <.001 | <.001 | <.001 | <.001 | <.001 | <.001 | <.001 | <.001 |
| History | −0.004 (–0.009 to 0.001) | −0.001 (–0.007 to 0.005) | 0.001 (–0.004 to 0.007) | 0.003 (–0.002 to 0.008) | .11 | .74 | .61 | .23 | .16 | .74 | .89 | .34 |
| Rank/100 | −0.020^e^ (−0.030 to −0.009) | −0.013^f^ (−0.026 to −0.001) | −0.007 (–0.018 to 0.004) | −0.010^g^ (–0.021 to 0.000) | <.001 | .04 | .18 | .06 | .001 | .08 | .37 | .11 |
| Tertiary | 0.366 (–0.470 to 1.203) | 0.426 (–0.554 to 1.406) | 0.110 (–0.745 to 0.965) | −0.224 (–1.058 to 0.611) | .39 | .39 | .80 | .60 | .45 | .45 | .99 | .64 |
| Private | 0.358 (–0.208 to 0.924) | 0.330 (–0.333 to 0.992) | 0.256 (–0.320 to 0.833) | 0.242 (–0.324 to 0.808) | .22 | .33 | .38 | .40 | .27 | .44 | .61 | .50 |
| General | −0.440^f^ (−0.793 to −0.087) | −0.091 (–0.504 to 0.323) | −0.248 (–0.609 to 0.113) | −0.456^f^ (−0.808 to −0.104) | .02 | .67 | .18 | .01 | .03 | .71 | .41 | .03 |
| ln(Income) | 1.944^e^ (0.520 to 3.368) | 1.406^g^ (–0.173 to 2.984) | 0.081 (–1.061 to 1.223) | 1.329^g^ (–0.217 to 2.874) | .007 | .08 | .89 | .09 | .02 | .14 | .89 | .16 |
| ln(density) | −0.271 (–0.613 to 0.072) | −0.284 (–0.667 to 0.098) | −0.021 (–0.307 to 0.265) | −0.303 (–0.671 to 0.064) | .12 | .15 | .89 | .11 | .16 | .21 | .95 | .17 |
| Age | −4.653 (–17.717 to 8.411) | −6.707 (–21.125 to 7.711) | 1.716 (–8.490 to 11.923) | −4.474 (–18.726 to 9.778) | .49 | .36 | .74 | .54 | .52 | .45 | .99 | .62 |
| Random effect ε_k_ | √ | √ | √ | √ | √ | √ | √ | √ | √ | √ | √ | √ |
| Constant | −16.206^e^ (−24.698 to −7.714) | −13.862^e^ (−23.243 to −4.482) | −4.810 (–11.587 to 1.967) | −12.751^e^ (−22.028 to −3.475) | <.001 | .004 | .16 | .007 | .001 | .01 | .44 | .02 |

^a^n=530.

^b^n=530.

^c^n=530.

^d^n=530.

^e^*P*<.01.

^f^*P*<.05.

^g^*P*<.10.
